# INTERASPIRE: an International Survey of Coronary Patients; Their Cardiometabolic, Renal and Biomarker Status; and the Quality of Preventive Care Delivered in All WHO Regions: In Partnership with the World Heart Federation, European Society of Cardiology, Asia Pacific Society of Cardiology, InterAmerican Society of Cardiology, and PanAfrican Society of Cardiology.

**DOI:** 10.1007/s11886-021-01568-2

**Published:** 2021-08-19

**Authors:** John William McEvoy, Catriona Jennings, Kornelia Kotseva, Guy De Backer, Dirk De Bacquer, Iris Erlund, Gregory Y H Lip, Kausik K Ray, Lars Rydén, Agnieszka Adamska, David A Wood

**Affiliations:** 1 Co-ordinating Centre: National Institute for Prevention and Cardiovascular Health, National University of Ireland Galway, Galway, Ireland; 2 Discipline of Medicine, Clinical Science Institute, National University of Ireland Galway, University Road, Galway, Ireland H91 TK33; 3 Department of Public Health and Primary Care, Ghent University, Ghent, Belgium; 4 Department of Government Services, Finnish Institute for Health and Welfare (THL), Helsinki, Finland; 5 Arrhythmia Centre: Liverpool Centre for Cardiovascular Science, University of Liverpool and Liverpool Heart & Chest Hospital Liverpool, Liverpool, UK; 6 Department of Clinical Medicine, Aalborg University, Aalborg, Denmark; 7 Imperial Centre for Cardiovascular Disease Prevention, Coordinating Centre for the FH Studies Collaboration: Department of Public Health and Primary Care, Imperial College, London, UK; 8 Diabetes Centre: Cardiology Unit, Department of Medicine Solna, Karolinska Institute, Stockholm, Sweden

**Keywords:** Cardiovascular, Secondary prevention, Hypertension, Lipids, Diabetes mellitus, Lifestyle

## Abstract

**Purpose of Review:**

To describe the INTERASPIRE scientific protocol—an international survey of secondary prevention of coronary heart disease (CHD).

**Recent Findings:**

This international survey is being conducted through National Societies of Cardiology in selected countries from each of the six WHO regions and has the following overall aims: (i) describe prevalence of cardiometabolic and renal risk factors together with biomarkers in CHD patients; (ii) describe current risk factor management through lifestyle changes and cardioprotective drug therapies; (iii) provide an objective assessment of clinical implementation of preventive care by comparison with the lifestyle and risk factor targets defined in international and national guidelines; (iv) investigate the reasons for variation in preventive cardiology practice between regions and countries; and (v) promote the principles of best preventive cardiology practice.

**Summary:**

This international survey will provide a unique picture of CHD patients; their cardiometabolic, renal and biomarker status; lifestyle and therapeutic management; and the quality of preventive care provided in all WHO regions.

## Introduction

The main objectives of cardiovascular (CVD) prevention are to reduce cardiovascular morbidity and mortality, improve quality of life and increase life expectancy. The 52-country INTERHEART study has shown that the classical risk factors for coronary heart disease (CHD) account for most of the risk of MI worldwide [1]. Therefore approaches to prevention can be based on similar principles worldwide, by targeting all these common risk factors, which have the potential to prevent most cases of premature myocardial infarction. The World Heart Federation Roadmap for secondary prevention of cardiovascular disease identifies roadblocks and potential solutions to improve cardiovascular health and help reach the target in the Sustainable Development Goals: achieve a 30% reduction in non-communicable diseases, principally by reducing CVD, by 2030 [2].

There is a wealth of scientific evidence supporting interventions in relation to lifestyle (smoking, diet and physical activity); the treatment of obesity, hypertension, dyslipidaemia and diabetes; and the selective use of prophylactic drug therapies. All these interventions can reduce cardiovascular morbidity and mortality in those with established atherosclerotic disease and can also reduce the risk of developing atherosclerotic disease.

This scientific evidence informs international, regional and national guidelines on CVD prevention, which define patient priorities for preventive action and lifestyle and treatment goals [3, 4•, 5•, 6•, 7–9]. Although there is common agreement between guidelines on which patients to prioritise for prevention, and the principle of total risk assessment, there are differences between regions and countries for some risk factor goals, especially for smoking, body weight, blood pressure, total and LDL cholesterol and the management of diabetes. The top priorities in all guidelines are patients with atherosclerotic disease: coronary artery disease, cerebral artery disease and peripheral artery disease.

Guideline implementation in Europe has been evaluated in five cross-sectional surveys called EUROASPIRE (European Action on Secondary and Primary Prevention by Intervention to Reduce Events). The first survey was in 1995–1996, and this was followed by the second, third, fourth and fifth surveys in 1999–2000, 2006–2007, 2012–2014 and 2016–2018, respectively, through the European Society of Cardiology (ESC) Euro Heart Survey/EuroObservational Research Programme [10–12]. The objective of each survey was to determine whether in real-world practice patients with coronary heart disease were achieving the standards set in the ESC CVD prevention guidelines and whether there were any changes over time in lifestyle, risk factor and therapeutic management [13, 14]. The fourth European survey of Cardiovascular Disease Prevention and Diabetes (EUROASPIRE IV) merged with the EuroHeart Survey on Diabetes Mellitus and incorporated an assessment of dysglycaemia (impaired fasting glycaemia (IFG), impaired glucose tolerance (IGT) and new diabetes) in all patients [15, 16]. The Vth EUROASPIRE survey continued this focus on cardiometabolic and renal disease in secondary prevention of 8261 coronary patients across 27 European countries [17•, 18•]. Outside Europe the largest international study of secondary prevention of CHD is the Prospective Urban Rural Epidemiological study (PURE), undertaken across 17 countries, which reports in 7519 coronary and stroke patients the dominance of unhealthy lifestyles and the inequitable use of secondary prevention drugs in patients with cardiovascular disease in high-, middle- and low-income countries [19, 20].

The INTERASPIRE survey began when the ESC EuroObservational Research Programme decided to expand the EUROASPIRE programme to include other WHO regions, starting with a pilot study in Malaysia and Argentina in 2019. The main survey (2020–2022) is now being organised in partnership with the World Heart Federation, Asia Pacific Society of Cardiology, InterAmerican Society of Cardiology and the Pan-African Society of Cardiology and includes selected countries in all 6 WHO regions: African Region, Region of Americas, Eastern Mediterranean Region, European Region, South-East Asia Region, and Western Pacific Region.

## Objectives

The specific objectives of the INTERASPIRE survey are:

1. To determine in patients with established CHD (acute coronary syndrome or revascularisation by angioplasty or coronary artery surgery) whether guidelines on cardiovascular disease prevention and rehabilitation are being followed
2. To compare diagnostic and therapeutic strategies in patients with established CHD in relation to glucose metabolism (impaired fasting glycaemia, impaired glucose tolerance and diabetes)
3. To compare diagnostic and therapeutic strategies together with risk prediction models for atrial fibrillation in patients with established CHD
4. To compare diagnostic and therapeutic strategies in prevalent cases of familial hypercholesterolaemia in patients with established CHD and the residual risk among these patients with current treatments
5. To compare diagnostic and therapeutic strategies in patients with established CHD in relation to chronic kidney disease (CKD)
6. To describe the prevalence of cardiovascular risk factors, acute and long-term cardiovascular complications and therapeutic management in patients with CHD with and without exposure to COVID-19
7. To measure new cardiovascular biomarkers to quantify residual risk and describe relationships with lifestyle, traditional risk factors and therapeutics and how these biomarkers impact outcomes
8. To follow-up all CHD patients 1 year after the interview for hospitalisations, cardiovascular procedures, cardiovascular events and cardiovascular and all-cause mortality and relate management to outcomes
9. To identify strategies for improving preventive care of CHD patients based on the INTERASPIRE survey results and make policy recommendations through WHF, continental and national societies of cardiology to WHO and national governments

## Study Design

An international survey of CHD patients is being conducted in selected countries from each of the six WHO regions around the world.

**Figure 1.**
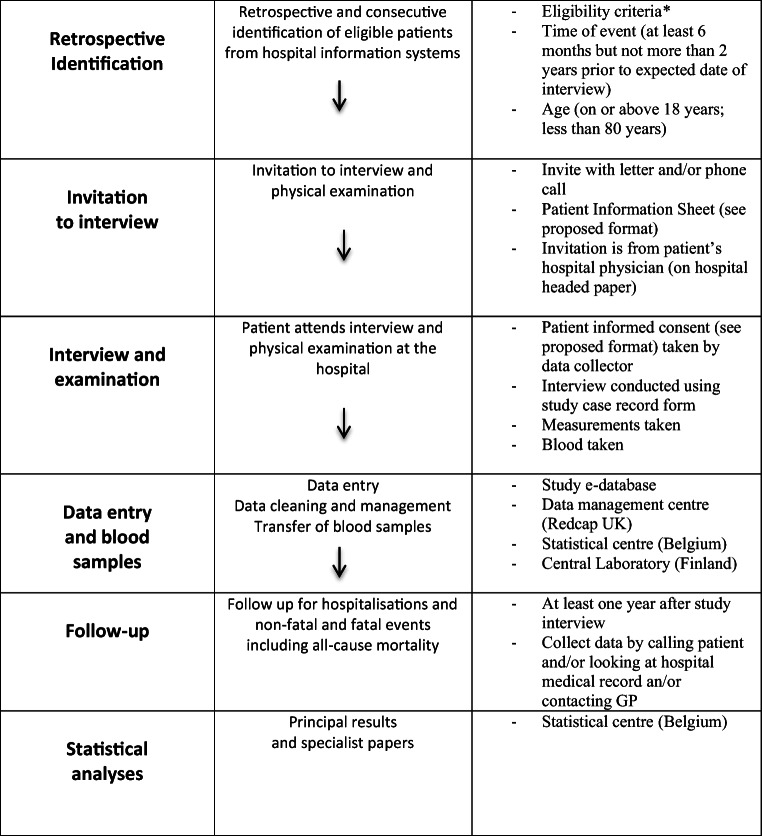
INTERASPIRE study flow chart. The single asterisk means elective CABG, elective PCI, acute coronary syndromes (acute myocardial infarction with ST elevation (STEMI) and Non-ST elevation MI (Non-STEMI) including those treated with primary PCI and/or CABG, and unstable angina).

In selected geographical regions within each country, and selected public hospitals, a consecutive sample of patients admitted with CHD is being identified retrospectively and information on their management recorded from their hospital medical record, including drugs on discharge. (See Figure 1 INTERASPIRE study flow chart).

Patients are then being interviewed and examined at least 6 months, but not later than 2 years, after their initial index hospital admission. Lifestyle is assessed together with standardised measurements of breath carbon monoxide, height, weight, abdominal circumference, blood pressure and central laboratory analysis of fasting lipids (total cholesterol, HDL cholesterol and triglycerides), fasting glucose, HbA1c, creatinine and cardiovascular biomarkers. Patients without a self-reported diagnosis of diabetes mellitus have an oral glucose tolerance test. All patients have urinary creatinine/albumin ratio measured in local laboratories.

All coronary patients are then followed up 1 year after interview for hospitalisations and cause specific and all-cause mortality.

## Study Population

WHO regions, selected countries and National Societies of Cardiology:

- **African Region**
  Kenya (Kenyan Cardiac Society)
  Nigeria (Nigerian Cardiac Society)
- **Regions of Americas**
  Argentina (Argentine Society of Cardiology)
  Colombia (Colombian Society of Cardiology and Cardiovascular Surgery)
  USA (American College of Cardiology)
- **Eastern Mediterranean Region**
  Egypt (Egyptian Society of Cardiology)
  Qatar (Gulf Heart Association-Qatar)
  United Arab Emirates (Emirates Cardiac Society)
- **European Region**
  Poland (Polish Cardiac Society)
  Portugal (Portuguese Society of Cardiology)
  Russia (Russian National Society of Preventive Cardiology)
- **South-East Asia Region**
  Indonesia (Indonesian Heart Association)
  Malaysia (National Heart Association of Malaysia)
  Philippines (Philippine Heart Association)
  Singapore (Singapore Heart Foundation/Singapore Cardiac Society)
- **Western Pacific Region**
  Australia (Cardiac Society of Australia and New Zealand)
  China (Chinese Cardiovascular Association)

### Geographical Areas and Hospital Sampling Frame

Within each country, at least three geographical areas are selected and all hospitals serving those populations identified. The areas include at least one hospital offering interventional cardiology and cardiac surgery and one or more acute hospitals receiving patients with an acute coronary syndrome. Hospitals are selected in each geographical area in such a way that any patient presenting within the area with acute symptoms of CHD, or requiring revascularisation in the form of percutaneous coronary intervention (PCI) or coronary artery bypass grafting (CABG), has an approximately equal chance of being included in the patient sample.

### Samples of Patients

Within each hospital, consecutive patients, men or women [≥18 years and <80 years at the time of identification], with first or recurrent clinical diagnosis or treatments for CHD (see below) are identified retrospectively from diagnostic registers, hospital discharge lists or similar sources between 6 months and 2 years before the expected date of interview. Patients may fulfil more than one of the following diagnostic criteria:

- Elective CABG.
- Elective PCI.
- Acute coronary syndromes (acute MI with ST elevation (STEMI) and Non-ST elevation MI (Non-STEMI) including those treated with primary PCI and/or CABG and unstable angina).

It is recognised that hospital diagnoses for acute MI, and unstable angina without evidence of infarction, may not always meet the World Health Organization (WHO) or other standard diagnostic criteria. However, it is important to include all cases diagnosed in each hospital as acute MI or unstable angina because, as a consequence of these diagnoses, they should have all received appropriate management for secondary prevention.

In order to achieve an interview response rate of at least 60%, a sufficient number of patients with CHD (approximately 660) are identified across all hospital sites to give a total sample of 400 interviewed coronary patients in each country.

## Training of Data Collectors

Training of data collectors from the pilot countries (Argentina and Malaysia) was conducted in person at the National Society headquarters by the coordinating team from NIPC. Following the advent of the COVID-19 pandemic in early 2020, the coordinating centre developed an online training platform using an open-source learning platform (https://moodle.org/). All other countries are now being trained using this online facility. All study documentation, manuals and training videos can be accessed on this platform.

### Data Collection

Trained research staff identify the patients, review medical records and interview and examine the patients using standardised methods and instruments.

The data collection takes place at least 6 months and at most 2 years after the date of the acute index hospital admission or procedure and is based on a retrospective review of patient medical records, a patient interview and examination, and then a 1-year follow-up for events and mortality.

### Review of Patient Medical Records

#### Patient Record Form

The following information is obtained from the medical records both prior to and following the date of acute index hospital admission or procedure:

- Personal and demographic details
- Past medical history, including hypertension, hyperlipidaemia, glucose metabolism and COVID-19 infection
- Family history of premature CHD in first-degree relatives and history of hypercholesterolaemia in first-degree relatives
- Recorded measurements of blood pressure, diabetes, lipids and smoking status (on treatment and, if known, pre-treatment levels)
- Diagnostic procedures undertaken (or scheduled): coronary angiography, echocardiography or other imaging modalities, exercise or pharmacological stress tests
- Measurements of weight, height, body mass index and waist circumference
- Presence of tendon xanthomata and corneal arcus
- Measurement of blood pressure
- Blood tests: lipids (total cholesterol, HDL- cholesterol, LDL-cholesterol, triglycerides), fasting/random plasma glucose, HbA1c, OGTT, eGFR/ serum creatinine
- Rhythm recorded on 12 lead ECG
- Urine albumin/creatinine ratio
- Medication (generic name and total daily dose) including antiviral and other medications being studied for COVID-19 and detail on ACEI/ARBs

### Patient Interview and Examination

#### Patient Interview Form

The following information is obtained at least 6 months and at most 2 years after the acute index admission or procedure:

- Personal and demographic details
- Personal cardiovascular history, including stroke, TIA (transient ischaemic attack), PAD (peripheral artery disease), heart failure, valvular heart disease and age at which disease was first manifest
- Other medical history, including hypertension, hyperlipidaemia, glucose metabolism and COVID-19 infection
- Family history of CHD for patients with premature disease (men < 55 years and women < 60 years) in first-degree relatives
- Reported lifestyle and other risk factor management in relation to smoking, diet (including weight reduction), alcohol, exercise, blood pressure, lipids and glucose and reported referral to and attendance at cardiac rehabilitation programs
- Medication (generic name and total daily dose) including detail on ACEI/ARBs
- Level of education, school attendance and employment status

The following examination and measurements are made:

- Height (calibrated measuring stick), weight (digital scales) and waist circumference (metal tape measure)
- Breath carbon monoxide (Bedfont Scientific, Model Micro^+^ Smokerlyzer)
- Presence of tendon xanthomata and corneal arcus
- Auscultation of the heart for systolic and diastolic murmurs
- Blood pressure (Omron M6 (HEM 7211-E) automatic digital sphygmomanometer)
- A digitised 12-lead electrocardiogram
- A cardiac ultrasound examination report from the medical record
- Venous blood processed for serum and plasma and stored at −80° at a country level and then transported on dry ice to the Central Laboratory in Helsinki to measure total cholesterol, HDL cholesterol, triglycerides, calculated LDL cholesterol, creatinine, HbA1c, COVID antibodies (SARS CoV-2 IgG, SARS CoV-2) and biomarkers: high-sensitivity Troponin I, brain natriuretic peptide (BNP), NT-pro-BNP, galectin-3 and potentially soluble urokinase-type plasminogen activator receptor (SuPAR)
- Local laboratory measurements of fasting glucose, 2-h post glucose load in an OGTT and urine for albumin/creatinine ratio
- Long-term storage of blood: serum, plasma and whole blood for HbA1c
- Self-administered questionnaires: HADS (Hospital Anxiety and Depression Scale), HeartQoL (health-related quality of life), EuroQoL (European Quality of Life Questionnaire EQ-5D) and Medication Adherence Questionnaire

### One-Year Follow-Up

Follow-up will be conducted at 1 year following the date of interview for vital status and procedures or events as listed below:

- Vital status: alive/dead or unknown
- Cause of death: CHD, stroke, other vascular, cancer, other cause, unknown
- Procedures or events performed since the date of interview:
- Hospitalisation for: PCI, CABG, AMI, stroke or TIA, heart failure

- Diagnosed with diabetes mellitus: yes, no, unknown

## Data Management Centre

Web-based data entry will be used at all sites and data management will be undertaken by the EURObservational Research Programme Department (Celine Arsac, EORP Team Manager; Clara Berle, Clinical Project Manager; and Gagan Chhabra, Data Manager) at the European Heart House, Sophia Antipolis, Nice, France, for the pilot study in Argentina and Malaysia and by AIMES Management Services Limited (Richard Spragg, Technical Director; Shaun Atkinson, Database Administrator) Liverpool, UK, for the main study.

## Laboratory Analyses

Central laboratory analysis of whole blood HbA1c, serum total cholesterol, HDL cholesterol, triglycerides and creatinine as well as fasting plasma glucose is undertaken in all coronary patients together with COVID-19 antibodies and cardiovascular biomarkers. OGTT from plasma and albumin/creatinine ratio from urine are analysed at each hospital site. Whole blood, serum and plasma are frozen locally and transferred to the central laboratory for biochemical analysis and long-term storage.

## Statistics

### Sample Size Calculations

In order to estimate prevalences with precision, a sample of 400 coronary patients attending interview is sufficient to estimate within-country prevalences with a precision of at least 5% and a confidence of 95%. The precision of estimates after stratification for age and gender within a country are limited but combining data from all countries will allow precise estimates by age and gender.

### Statistical Analysis

All the patients enrolled are included in the analyses. Since this is an observational study, descriptive summaries will be presented for all the patients and for subgroups of patients to report the prevalence of risk factor recording and management within and between countries. Statistical tests may be carried out for exploratory purposes, as appropriate. Multivariable analyses may be used to explore relationships between baseline covariates and post-baseline endpoints, as appropriate.

## Outcome Measures

The main outcomes of interest include the proportions of hospital coronary patients achieving lifestyle, risk factors and therapeutic targets for cardiovascular disease prevention. The management of risk in terms of lifestyle intervention and the use of drug therapies will be evaluated in relation to the lifestyle and therapeutic goals defined in the guidelines on cardiovascular disease prevention:

- Smoking habit (self-reported and validated with breath carbon monoxide (CO) (Bedfont Scientific, Model Micro ^+^ Smokerlyzer)
- Diet (self-reported questions)
- Physical activity (self-reported questions and the validated Godin Leisure time exercise questionnaire)
- Overweight/obesity
  Height and weight (calibrated measuring stick and digital scales)
  Waist circumference (metal tape measure)
  Body mass index (BMI)
- Diabetes (self-reported) and new diabetes, impaired fasting glycaemia and impaired glucose tolerance (oral glucose tolerance test using HemoCue or glucose analysis by the local hospital laboratory) and HbA1c
- Blood pressure will be measured twice in a sitting position on the right upper arm and the mean of the two measurements will be used in the data analyses. (Omron M6 (HEM 7211-E) automatic digital sphygmomanometer)
- Serum total cholesterol, high-density lipoprotein (HDL) cholesterol, triglycerides, calculated low-density lipoprotein (LDL) cholesterol), serum creatinine, plasma glucose, HbA1c, COVID-19 antibodies and biomarkers: high-sensitivity Troponin I, brain natriuretic peptide (BNP), NT-pro-BNP, galectin-3 and potentially soluble urokinase-type plasminogen activator receptor (SuPAR)
- Variables for the Dutch Lipid Clinic Network criteria to estimate the probability of FH, including personal/family history/laboratory measurements/physical examination and presence or absence of a molecular diagnosis
- Atrial fibrillation
- Urine for albumin/creatinine ratio
- Drug therapies:
  Antithrombotics
  Beta-blockers
  ACE inhibitors
  Angiotensin-II receptor antagonists
  Lipid-lowering drugs
  Hypoglycaemic treatments
  Diuretics
  Calcium channel blockers
  Nitrates
  If inhibitors
  Metabolic agents
  Nicotine replacement therapy
  Bupropion hydrochloride
  Varenicline
  Antiobesity drugs
  Antidepressants
  Antianxiety drugs
- Psycho-social measures (self-reported questions and the validated HADS, EQ-5D and HeartQol)

Finally, we will ascertain 1-year outcomes, specifically cardiovascular events (non-fatal coronary and cardiovascular events, including revascularisation and hospitalisations), and cardiovascular/total mortality

## Conclusion

This international survey of secondary prevention of CHD patients in selected countries across all WHO regions will provide an in-depth analysis of lifestyle, risk factor and therapeutic management within, and between, countries and regions in relation to international and national cardiovascular prevention guidelines. The arrival of the COVID-19 pandemic led to a rapid change to the scientific protocol to capture exposure to this new virus, by measuring antibodies, which can then be related to subsequent cardiovascular outcomes. The results of this survey will be made available to the World Heart Federation Global Observatory, and also to National Societies of Cardiology, to inform advocacy to policy makers in WHO and national governments to raise standards of preventive cardiology.
